# Retrorectal Schwannomas: Atypical Presentation and Controversial Surgical Management

**DOI:** 10.1155/2021/5535283

**Published:** 2021-05-07

**Authors:** Wided Trimech, Farouk Ennaceur, Hiba Ben Hassine, Hanen Zneti, Faouzi Noomen, Khadija Zouari

**Affiliations:** Department of General and digestive Surgery, University Hospital Fattouma Bourguiba of Monastir, University of Monastir, Tunisia

## Abstract

Schwannomas also known as neurilemomas are benign tumors. Retrorectal schwannomas are extremely rare, accounting for 1 to 5% of all schwannomas. They are mostly asymptomatic but may present with symptoms such as pelvic pain, back pain, lower extremities pain, or constipation. Physical examination is often poor. Imaging (CT, MRI) and fine needle biopsy can often help orient the diagnosis. The treatment of choice is monoblock resection of the mass. The prognosis is good. Recurrence has been reported especially after intralesional enucleation. We report a case of a 41-year-old male patient consulting for chronic low back pain eventually diagnosed with retrorectal schwannoma. We performed a surgical resection and the histological examination was consistent with the diagnosis of benign (ancient) schwannoma.

## 1. Introduction

Schwannomas also known as neurilemomas are benign tumors [1]. They derive from the neoplastic transformation of Schwann cells of the peripheral nerve sheath [2]. Malignant change is uncommon, yet malignant tumors associated with von Recklinghausen's disease have been reported [1]. Retrorectal schwannomas are extremely rare as they represent only 1 to 5% of all schwannomas [3]. These tumors are found most commonly in the head, neck, and extremities, while only 0.5–5% occur in the retrorectal space [4], representing 1 in 250 of all retrorectal tumors [5]. They can be asymptomatic and incidentally revealed by a pelvic or a rectal examination; however, they can be revealed by pelvic pain, back pain, lower extremities pain, or constipation [3, 5]. Imaging tests can be suggestive of schwannomas and help to identify the exact location of the mass. A biopsy of such a lesion is controversial but if done it can differentiate between benign and malignant tumors [6]. The treatment of choice is local monoblock resection of the mass [5]. Hereby, we report a case of a male adult consulting for chronic low back pain who was eventually diagnosed with retrorectal schwannoma.

## 2. Case Report

**Figure 1:**
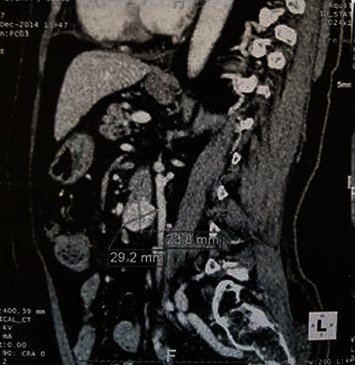
CT aspect of the tumor.

**Figure 2:**
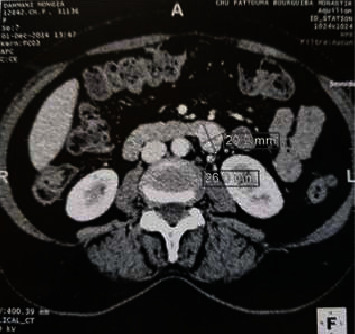
CT aspect of the tumor.

A 41-year-old man complaining of pelvic pain and bilateral sciatica radiating towards the right inguinal and lumbar area for 2 years. He reported some abdominal discomfort but no urinary or neurologic symptoms were reported. Abdominal examination was poor. Rectal examination found a 3.5 cm smooth, fixed, retrorectal mass. Computed tomography revealed a 29.2 × 26 mm solid, heterogeneous pelvic mass with well-defined limits containing small calcifications (Figures 1 and 2). A CT-guided biopsy was performed but was inconclusive. Magnetic resonance (MR) imaging showed a 3 cm with low signal on T1-weighted image, with a high signal intensity and heterogeneous signal intensity on T2-weighted image.

**Figure 3:**
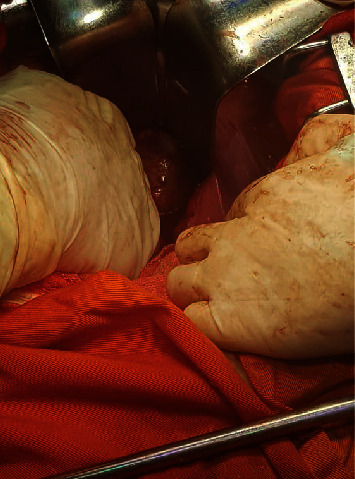
Preoperative aspect of the tumor.

Laparotomy was performed using a midline incision. A 3 cm encapsulated mass was found (Figure 3), and it was adherent to the right pelvic sidewall and the periosteum of the sacrum. The bladder, prostate, seminal vesicle, and rectosigmoid colon were carefully dissected free, and we performed a monoblock resection of the tumor. The follow-up was simple and the patient was discharged 3 days later.

Histological examination showed benign schwannian cells, along with an alternating Antoni A and Antoni B pattern, and areas of nuclear atypia were noted. The specimen tested positive for S100 and had one mitotic figure per 30 high-powered fields (HPF), all consistent with the diagnosis of benign (ancient) schwannoma. The patient has remained asymptomatic, with no sign of recurrence on CT scan at 36 months of follow-up.

## 3. Discussion

The retrorectal space is limited anteriorly by the rectum, by the sacrum posteriorly, the ureters, iliac vessels, and sacral nerve roots on each side, it extends superiorly to the peritoneal reflection superiorly and the levator musculature inferiorly [7, 8]. A variety of tumors can be found in this space arising from adjacent tissues (vessels, peripheral nerves of sympathetic and parasympathetic nervous systems, and connective tissue) [8]. Schwannomas are encapsulated slow-growing painless neurogenic benign tumors associated with von Recklinghausen's disease in 18% of cases. These tumors occur without predominance of sex in the 20- to 50-year age group [1]. Because of their location, deep schwannomas such as prerectal schwannomas usually reach a large size before becoming symptomatic, and it is estimated that patient can be asymptomatic for 7 years [3, 5]. Accompanying symptomatology is often nonspecific, including pain or pelvic mass syndrome and signs of compression of neighboring organs such as constipation and low back pain [3], which is the case with our patient. During Physical examination, signs such as “café au lait” spots, freckling of the axillae, or inguinal regions should be searched since they may be present in VRH syndrome [3]. Digital rectal examination reveals an intact rectal mucosa with a retrorectal firm, regular, insensitive mass. Preoperative diagnosis of retrorectal schwannomas is a challenge for doctors. Regarding the wide diversity of tumors in this region, a biopsy can be useful to differentiate between benign and malignant tumors and to indicate the extent of resection based on histological analysis including immunohistochemistry [6, 9]. But such a procedure is still controversial since complications such as infection or hemorrhage and even tumor dissemination remain possible [1, 7]. When a biopsy is performed, as is the case with our patient, it is usually done under CT guidance but it is not always conclusive. In most schwannomas, anatomopathological examination shows a well-defined mass surrounded by a capsule. The pathognomonic element of a neurilemoma is the coexistence of Antoni A areas composed of compact spindle cells and Antoni B areas which are remarkably less cellular [10]. Imaging findings can be suggestive of schwannomas, but they are similar to those found with neurofibromas. CT shows a well-limited mass hypo or isodense with an enhancement after contrast injection. Unlike neurofibromas, schwannomas commonly display a nonenhancement of necrotic or cystic zones [10]. MRI scan is the gold standard in investigating a retrorectal tumor with a positive predictive value of 100% for malignancy in some series [8]. On T1-weighted images, schwannomas have intermediate signal intensity, and on T2-weighted images, schwannomas show increased signal intensity with a uniform enhancement for small schwannomas and a heterogenic enhancement for large ones. The capsule can be visualized as a low-intensity rim in 70% of cases [10]. The optimal treatment for retrorectal tumors in particular schwannomas is complete en bloc surgical excision [5, 8]. Tumors arising above S3 require anterior laparotomy while tumors arising below S3 require a perineal approach [3, 11]. Some papers reported laparoscopic techniques being applied to such tumors with better preoperative view, less postoperative pain, and shorter hospital stay but with difficulties during the dissection of the lower pole of the tumor [11, 12]. In the case of a retrorectal schwannoma, the overall survival is around 100%. Recurrence rates may vary depending on the resection limit; in fact, it ranges from 16% to 54% after intralesional enucleation [13].

## 4. Conclusion

Symptoms can exceptionally reveal a retrorectal schwannoma. Physical examination is often poor. Imaging (CT, MRI) and fine needle biopsy can often orient the diagnosis. Monoblock resection remains the treatment of choice. The prognosis is good. Recurrence has been reported specially after intralesional enucleation.
